# Machine learning-based prediction model for late recurrence after surgery in patients with renal cell carcinoma

**DOI:** 10.1186/s12911-022-01964-w

**Published:** 2022-09-13

**Authors:** Hyung Min Kim, Seok-Soo Byun, Jung Kwon Kim, Chang Wook Jeong, Cheol Kwak, Eu Chang Hwang, Seok Ho Kang, Jinsoo Chung, Yong-June Kim, Yun-Sok Ha, Sung-Hoo Hong

**Affiliations:** 1 Department of Medical Informatics, College of Medicine, The Catholic University of Korea, Seoul, 06591 Korea; 2 Department of Biomedicine and Health Sciences, College of Medicine, The Catholic University of Korea, Seoul, 06591 Korea; 3 Department of Urology, Seoul National University College of Medicine, Seoul National University Bundang Hospital, Seongnam, 13620 Korea; 4 Department of Urology, Seoul National University College of Medicine, Seoul National University Hospital, Seoul, 03080 Korea; 5 Department of Urology, Chonnam National University Medical School, Gwangju, 61469 Korea; 6 Department of Urology, Korea University School of Medicine, Seoul, 02841 Korea; 7 Department of Urology, National Cancer Center, Goyang, 10408 Korea; 8 Department of Urology, Chungbuk National University College of Medicine, Cheongju, 28644 Korea; 9 Department of Urology, College of Medicine, Chungbuk National University, Cheongju, 28644 Korea; 10 Department of Urology, Kyungpook National University Chilgok Hospital, School of Medicine, Kyungpook National University, Daegu, 41404 Korea; 11 Department of Urology, Seoul St. Mary’s Hospital, College of Medicine, The Catholic University, Seoul, 06591 Korea

**Keywords:** Renal cell carcinoma, Machine learning, ROC curve: KOrean Renal Cell Carcinoma, Late recurrence

## Abstract

**Background:**

Renal cell carcinoma is characterized by a late recurrence that occurs 5 years after surgery; hence, continuous monitoring and follow-up is necessary. Prognosis of late recurrence of renal cell carcinoma can only be improved if it is detected early and treated appropriately. Therefore, tools for rapid and accurate renal cell carcinoma prediction are essential.

**Methods:**

This study aimed to develop a prediction model for late recurrence after surgery in patients with renal cell carcinoma that can be used as a clinical decision support system for the early detection of late recurrence. We used the KOrean Renal Cell Carcinoma database that contains large-scale cohort data of patients with renal cell carcinoma in Korea. From the collected data, we constructed a dataset of 2956 patients for the analysis. Late recurrence and non-recurrence were classified by applying eight machine learning models, and model performance was evaluated using the area under the receiver operating characteristic curve.

**Results:**

Of the eight models, the AdaBoost model showed the highest performance. The developed algorithm showed a sensitivity of 0.673, specificity of 0.807, accuracy of 0.799, area under the receiver operating characteristic curve of 0.740, and F1-score of 0.609.

**Conclusions:**

To the best of our knowledge, we developed the first algorithm to predict the probability of a late recurrence 5 years after surgery. This algorithm may be used by clinicians to identify patients at high risk of late recurrence that require long-term follow-up and to establish patient-specific treatment strategies.

**Supplementary Information:**

The online version contains supplementary material available at 10.1186/s12911-022-01964-w.

## Background

Renal cell carcinoma (RCC) accounts for approximately 90% of all renal malignancies; therefore, cancer of the kidney is commonly referred to as RCC. Kidney cancer is the ninth most common cancer in men, with an estimated 431,288 new cases and 179,368 cancer deaths worldwide in 2020 [1]. According to the Korea National Cancer Center statistics in 2018, the incidence of kidney cancer steadily increased from 1999 to 2018 [2]. However, these statistics also show that the incidence of RCC is not high compared with other cancers, but the mortality rate of RCC increased by 3.92% per year between 1975 and 2009 [3], and RCC is the second most lethal urologic malignancy [4].

Although other alternative treatments exist, surgery is the gold standard treatment according to guidelines [5–7]. Though radical nephrectomy is the primary treatment, recently, partial nephrectomy has been used to preserve kidney function [8]. However, despite these treatments, cancer recurs in 20–40% of patients [9–11]. Most cases of RCC recurrence occur within 5 years of surgery, while 10% of cases recur after 5 years [12–14]. Therefore, continuous follow-up after surgery is essential for RCC patients.

One of the biological characteristics of RCC is late recurrence, which occurs 5 years after surgery [12]. Studies have been conducted to identify various factors that can significantly influence late recurrence in RCC [15–18]. However, these studies, which relied on traditional statistical methods such as logistic regression analysis and the Cox proportional-hazards model to identify risk factors, have not led the ability to accurately predict late recurrence of renal cell carcinoma.

Recently, with the development of computer technology, studies that apply machine learning and deep learning methods to large-capacity data, including medical fields, are being actively conducted [19–23]. In the medical field, machine learning has shown excellent performance when applied to cancer diagnosis [24–26]. If data are used by various machine learning techniques that combine and analyze the risk factors discovered in these studies, it is possible to predict late recurrence. In two previous studies, machine learning models for predicting early recurrence of RCC, within 5 years, showed good predictive performance with the area under the receiver operating characteristic curve (AUROC) values of 0.836 [27] and 0.840 [28], respectively. However, few studies have aimed to predict the late recurrence of RCC using machine learning techniques because the long-term follow-up of patients is challenging, late recurrence rate is only approximately 10%, and collecting enough data for analysis requires a long time.

In a previous study, data on patients with late recurrence was insufficient, and an algorithm to predict the probability of recurrence within 5 and 10 years was developed accordingly, although without distinguishing between early and late recurrence [27]. However, previous studies have demonstrated that the factors influencing early and late recurrence vary [12, 16]. Therefore, it is necessary to develop a model that is specifically designed for the accurate prediction of late recurrence. In addition, since late recurrence is very rare, it is difficult to track and collect case data within only a single institution.

In the present study, we developed an algorithm, using machine learning techniques to predict late recurrence after surgery using data of patients with RCC continuously collected from multiple institutional hospitals.

To the best of our knowledge, this is the first study to develop a model that predicts only late RCC recurrence that occurs 5 years after surgery. The algorithm was intended to help select patients at high risk of late recurrence for continuous monitoring to enhance early detection and appropriate treatment.

## Methods

### Study population

The KOrean Renal Cell Carcinoma (KORCC) web-based database system was established to collect the data on basic demographic and clinicopathological characteristics of patients with RCC in Korea [29]. Eight hospitals participating in the KORCC study group contributed to an established large cohort of patients with RCC by adding all consecutive patients from 1990 to date. This database construction project has been approved by the Seoul National University Bundang Hospital Ethics Committee (IRB No.: B1202/145-102). We collected data regarding 9,598 patients with RCC and 205 variables from the KORCC database and performed data preprocessing according to our study protocol. Variables include demographic and clinicopathological characteristics. To protect patients' personal information, resident registration numbers and hospital numbers have been excluded. Detailed variable types and distributions can be viewed through database construction studies [29]. This study protocol was approved by the Institutional Review Board (IRB) of the Catholic University of Korea (IRB No. KC20ZIDI0966). Informed consent was waived by the IRB of Catholic University of Korea since this study was retrospective and blinding of the personal information in the data was performed. The present study was designed and conducted in accordance with the relevant guidelines and regulations of the ethical principles for medical research involving human subjects, as stated by the World Medical Association Declaration of Helsinki.

### Variable selection

The following two-step process was performed to select the variables affecting late recurrence among the 205 variables. First, variables with a significant difference (P < 0.05) were selected between the non-recurrence and late recurrence groups using a t-test for continuous variables and a chi-squared test for categorical variables using statistical methods. In the first process, 18 variables were extracted as significant variables (see additional table in Additional File 1), and in the second process, seven clinically significant variables were selected based on the advice of urologists. The seven variables selected were tumor size, operation type, histologic type, operative methods, pathological tumor stage, pathological node stage, and lymphovascular invasion. A urologist with extensive experience in RCC surgery selected the final variable based on previous studies [15–18] and their clinical experience with following patients after surgery.

### Data screening

We included 9397 patients who underwent surgical treatment out of a total of 9598 patients with RCC to construct a dataset for the analysis. Among the 9397 patients, 4240 patients with a follow-up period of fewer than 5 years, 1037 patients with early recurrence, and 1164 patients with missing values were excluded. The 2956 patients who remained consisted of 2767 patients without recurrence and 189 patients with late recurrence over a follow-up of more than 5 years.

### Data splitting and SMOTE technique for imbalanced datasets

To train and evaluate the model, we split the data into two datasets: 70% for training and 30% for testing. In the training and test data, there were 140 (6.8%) and 49 (5.5%) patients with late recurrence, respectively, which was very low compared with the number of patients with non-recurrence (Table 1). Data imbalance is one of the problems in medical data analysis, and it occurs because the proportion of patients with specific cancer or disease is relatively small compared with normal patients in data collected at hospitals. Oversampling [30], undersampling [30], and synthetic minority oversampling technique (SMOTE) [31] are used as representative methods to resolve data imbalance. However, in oversampling, an overfitting problem occurs because of data duplication, and in undersampling, a large amount of information is lost [32]. SMOTE is also an oversampling method, but it solves imbalance by generating synthetic data rather than duplicating the data [31]. SMOTE has been previously applied to medical data with data imbalance problems [33, 34]. Given the data imbalance in the our study, we used the SMOTE technique to increase the late recurrence group to 50% of the training data, which resulted in a 1:1 ratio between the two groups (Table 1).

**Table 1:** Distribution of datasets before and after SMOTE application

	Training set (n = 2069)		Test set (n = 887)	
	Late recurrence group	Non-recurrence group	Late recurrence group	Non-recurrence group
Before	140 (6.8%)	1929 (93.2%)	49 (5.5%)	838 (94.5%)
After	1929 (50.0%)	1929 (50.0%)	49 (5.5%)	838 (94.5%)

### Model development and validation

We developed a model by applying eight representative machine learning techniques that showed excellent performance in classification problems in our dataset. The machine learning techniques used were support vector machine (SVM) [35], logistic regression [36], k-nearest neighbor (KNN) [37], naïve Bayes (NB) [38], random forest [39], gradient boost [40], AdaBoost [41], and extreme gradient boosting (XGBoost) [42]. We used a grid search algorithm [43] to optimize the hyperparameters used in the machine learning models, and in this case, we searched for optimal hyperparameters through 3-fold cross-validation to avoid overfitting. We measured the performance of each machine learning model in the hyperparameter obtained through the grid search and cross-validation. For validation, we calculated five parameters as follows: sensitivity, specificity, accuracy, AUROC, F1-score, and confusion matrix. The calculation method was as follows:

$${\text{Sensitivity}},\;{\text{Recall}} = \frac{{{\text{TP}}}}{{{\text{TP}} + {\text{FN}}}},$$

$${\text{Precision}} = { }\frac{{{\text{TP}}}}{{{\text{TP}} + {\text{FP}}}},$$

$${\text{Specificity}} = \frac{{{\text{TN}}}}{{{\text{TN}} + {\text{FP}}}},$$

$${\text{Accuracy}} = \frac{{{\text{TP}} + {\text{TN}}}}{{{\text{TP}} + {\text{FP}} + {\text{TN}} + {\text{FN}}}},$$

and

$${\text{F}}1{\text{ - score}} = { }\frac{2 \times Precision \times Recall}{{Precision + Recall}}$$

where TP is the number of true late recurrences, TN is the number of true non-recurrences, FP is the number of false-positive late recurrences, and FN is the number of false non-recurrences.

**Fig. 1:**
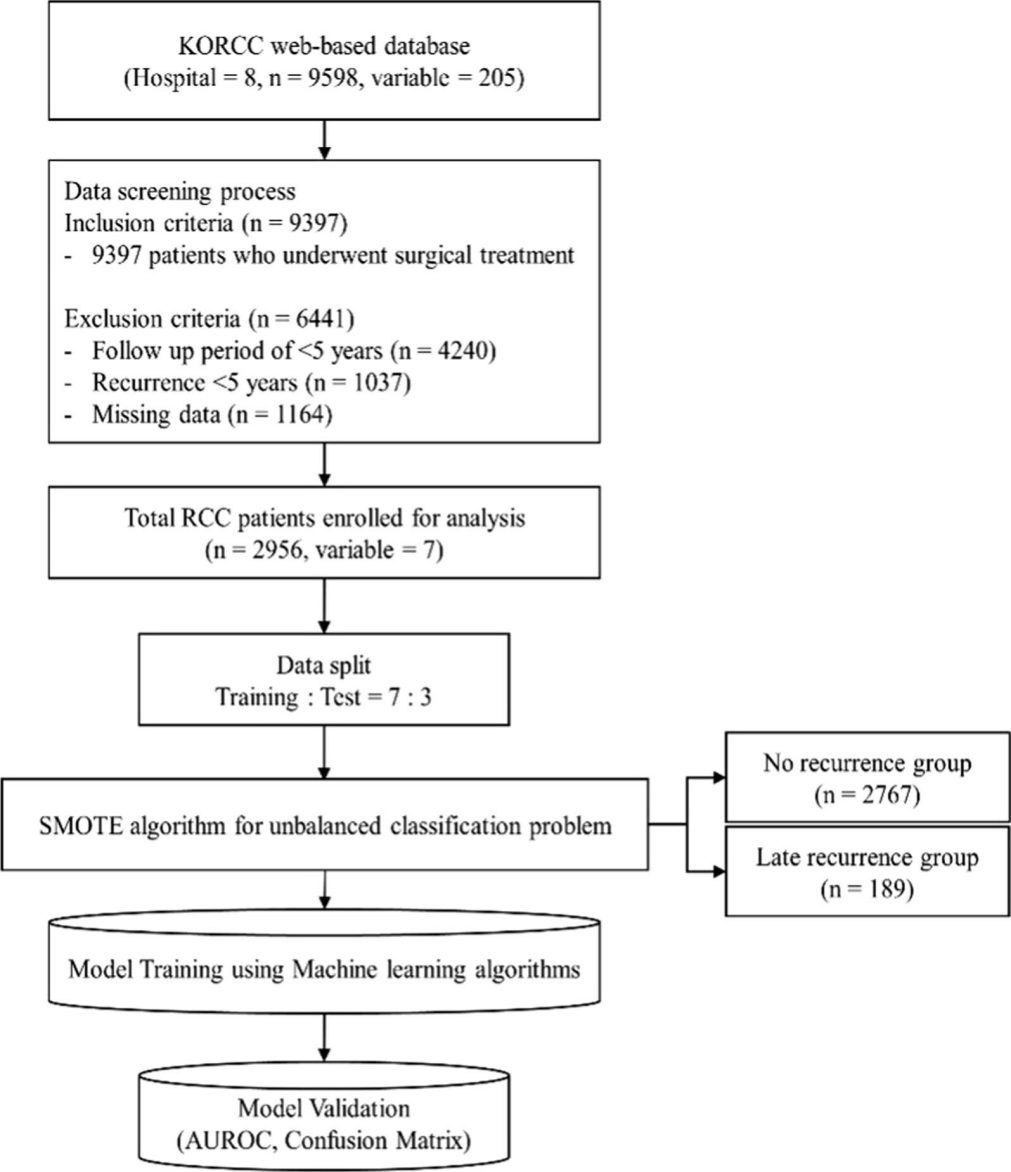
Overall process of development of the late recurrence prediction model

For the final model, the model with the highest performance was selected based on the AUROC. In addition, TP, NP, FP, and NP were confirmed from the results of applying the test data to the final selected model through the confusion matrix. The entire process from variable selection to model development and validation is shown in Fig. 1. We used Python (version 3.7.6) for statistical analysis and algorithm development.

## Results

### Patient characteristics

Table 2 shows the characteristics of the late recurrence and non-recurrence groups. The seven selected variables showed significant differences between the two groups. Regarding the operation type, the rate of partial nephrectomy was 51.7% in the non-recurrence group, which was slightly higher than that of radical nephrectomy (48.3%), but in the late recurrence group, the rate of radical nephrectomy was 82.0%, which was much higher than that of partial nephrectomy. Regarding the operative method, open and laparoscopic surgeries were similar at 44.1% and 38.0%, respectively, in the non-recurrence group, but in the late recurrence group, the rate of open surgery was notably high at 75.1%. The pathological tumor stages of 1a and 1b accounted for 86.3% of the total in the non-recurrence group, but 53.4% in the late recurrence group, and the remaining 46.6% were distributed in stages 2–4. The distributions of the remaining variables are presented in Table 2.

**Table 2:** Baseline characteristics of the patients with RCC

Variable	Late recurrence group (189 Patients)	Non-recurrence group (2767 Patients)	*P*-value
Operation type			< 0.001
Radical nephrectomy	155 (82.0%)	1336 (48.3%)	
Partial nephrectomy	34 (18.0%)	1431 (51.7%)	
Operative method			< 0.001
Laparoscopic	29 (15.3%)	1051 (38.0%)	
HALS	6 (3.2%)	78 (2.8%)	
Open	142 (75.1%)	1221 (44.1%)	
Robotic	12 (6.3%)	417 (15.1%)	
Pathological tumor stage			< 0.001
1a	52 (27.5%)	1811 (65.4%)	
1b	49 (25.9%)	579 (20.9%)	
2a	38 (20.1%)	131 (4.7%)	
2b	7 (3.7%)	48 (1.7%)	
3a	34 (18.0%)	167 (6.0%)	
3b	7 (3.7%)	20 (0.7%)	
3c	0 (0.0%)	2 (0.1%)	
4	2 (1.1%)	9 (0.3%)	
Pathological node stage			0.005
Nx	67 (35.4%)	1585 (57.3%)	
N0	115 (60.8%)	1169 (42.2%)	
N1	7 (3.7%)	13 (0.5%)	
Histologic type			0.002
Clear cell	172 (91.0%)	2345 (84.7%)	
Papillary	5 (2.6%)	41 (1.5%)	
Chromophobe	4 (2.1%)	222 (8.0%)	
Collecting duct	0 (0.0%)	3 (0.1%)	
Etc	8 (4.2%)	156 (5.6%)	
Lymphovascular invasion			< 0.001
No	172 (91.0%)	2701 (97.6%)	
Yes	17 (9.0%)	66 (2.4%)	
Tumor size (mm)	68.2$\pm 68.9$	38.8$\pm 27$.6	< 0.001

*HALS* hand-assisted laparoscopic surgery

### Hyperparameter optimization

We performed hyperparameter optimization for each machine learning model and used a grid search algorithm and cross-validation. The optimal parameter combinations were determined by exploring various hyperparameters, and the selected hyperparameters for each machine learning model are listed in Table 3. We first split the train dataset into three parts. Next, because hyperparameters found in unsplit datasets can cause overfitting problems, we compared the performance of the hyperparameters in Table 3 through grid search

**Table 3:** Hyperparameter optimization using the grid search algorithm

Algorithms	Hyperparameters
Kernel SVM	kernel: (linear, rbf*) C: (0.01, 0.1, 1*) gamma: (0.01, 0.05, 0.1, 0.5*, 5, 10)
Logistic regression	Penalty: (L1, L2*) C: (0.001, 0.01, 0.1, 1, 10*, 100)
KNN	n-neighbors: (2,4*,6,8,10)
Naïve Bayes	alpha: (0, 0.1, 1*, 5, 10, 20, 30)
Random forest	n_estimators: (10, 50, 100, 150, 200*) max_depth: (4, 8, 12, 16*,20)
Gradient boost	n_estimators: (10, 100, 200, 500*,1000) learning_rate: (0.05*, 0.01, 0.005, 0.001) max_depth: (1,3*, 6, 9, 12)
AdaBoost	n_estimators: (10, 100, 200, 500*, 1000) learning_rate: (0.05*, 0.01, 0.005, 0.001)
XGBoost	n_estimators: (10, 100, 200, 500, 1000*) learning_rate: (0.05*, 0.01, 0.005, 0.001) max_depth: (1*, 3, 6, 9, 12)

Penalty: Specify the norm used in the penalization (L1 = L1 regularization, L2 = L2 regularization); C, inverse of regularization strength; n-neighbors, number of neighbors; alpha, additive smoothing parameter (0 for no smoothing); n_estimators, number of trees; max_depth, maximum depth of the tree

*SVM* support vector machine, *KNN* k-nearest neighbour, *XGBoost* extreme gradient boosting

*Parameter finally selected through parameter optimization

### Model performance in predicting late recurrence of RCC

Performance was measured in parameters optimized for each machine learning model, and the results of the comparisons of sensitivity, specificity, accuracy, and AUROC are shown in Table 4. The model with the highest performance based on AUROC was AdaBoost. AdaBoost showed a predictive performance of 0.673 sensitivity, 0.807 specificity, 0.799 accuracy, 0.74 AUROC, and 0.609 F1-score.

**Table 4:** Performance of the machine learning algorithms

Model	Sensitivity	Specificity	Accuracy	AUROC	F1-score
Kernel SVM	0.551	0.852	0.835	0.702	0.579
Logistic regression	0.653	0.802	0.793	0.727	0.599
KNN	0.408	0.881	0.855	0.644	0.587
Naïve Bayes	0.612	0.805	0.795	0.709	0.566
Random forest	0.490	0.834	0.815	0.662	0.566
Gradient boost	0.531	0.868	0.849	0.699	0.576
AdaBoost	0.673	0.807	0.799	0.740	0.609
XGBoost	0.633	0.807	0.797	0.720	0.587

**Fig. 2:**
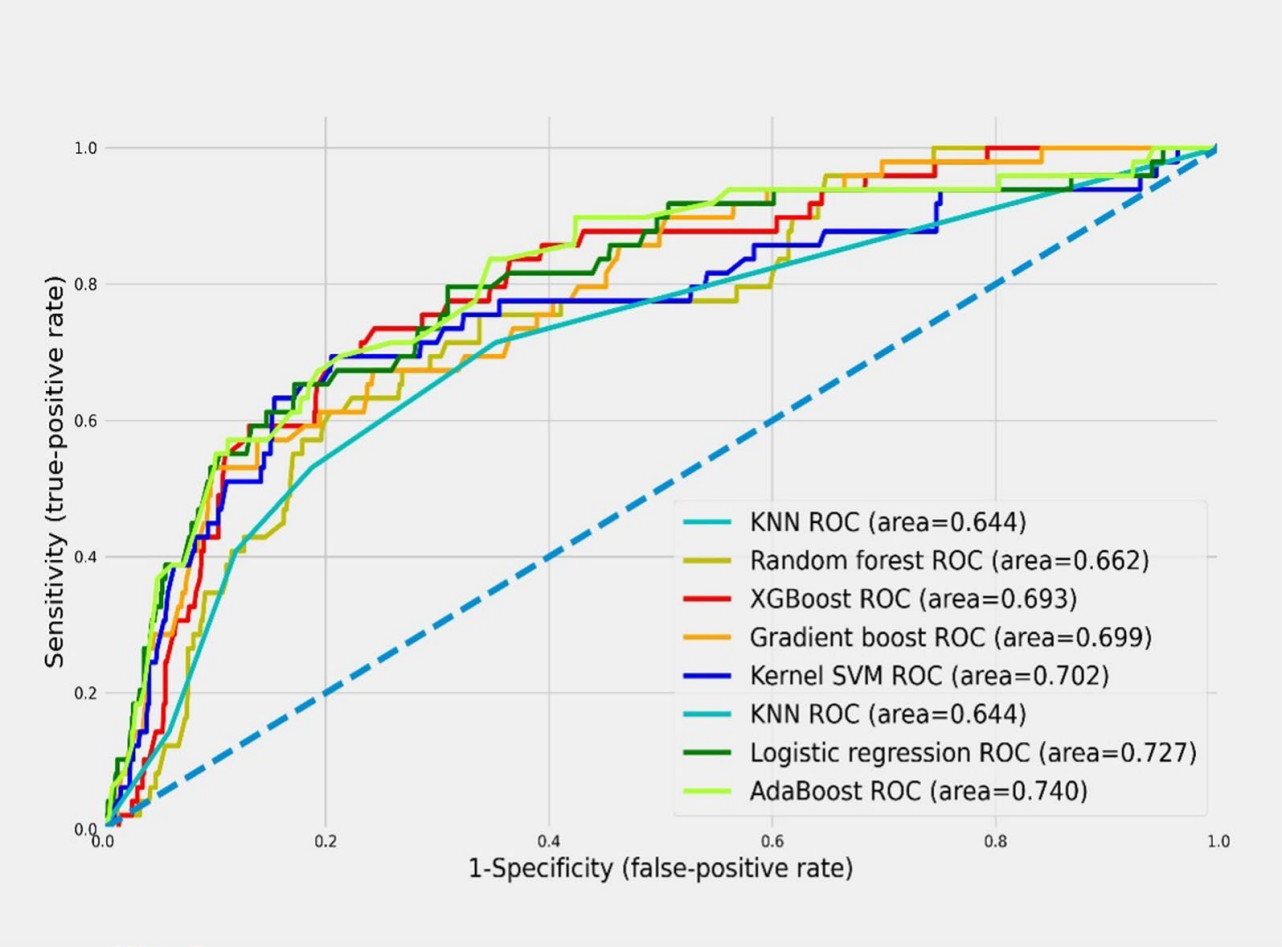
Receiver operating characteristic curve of machine learning models for predicting late recurrence after surgery

**Fig. 3:**
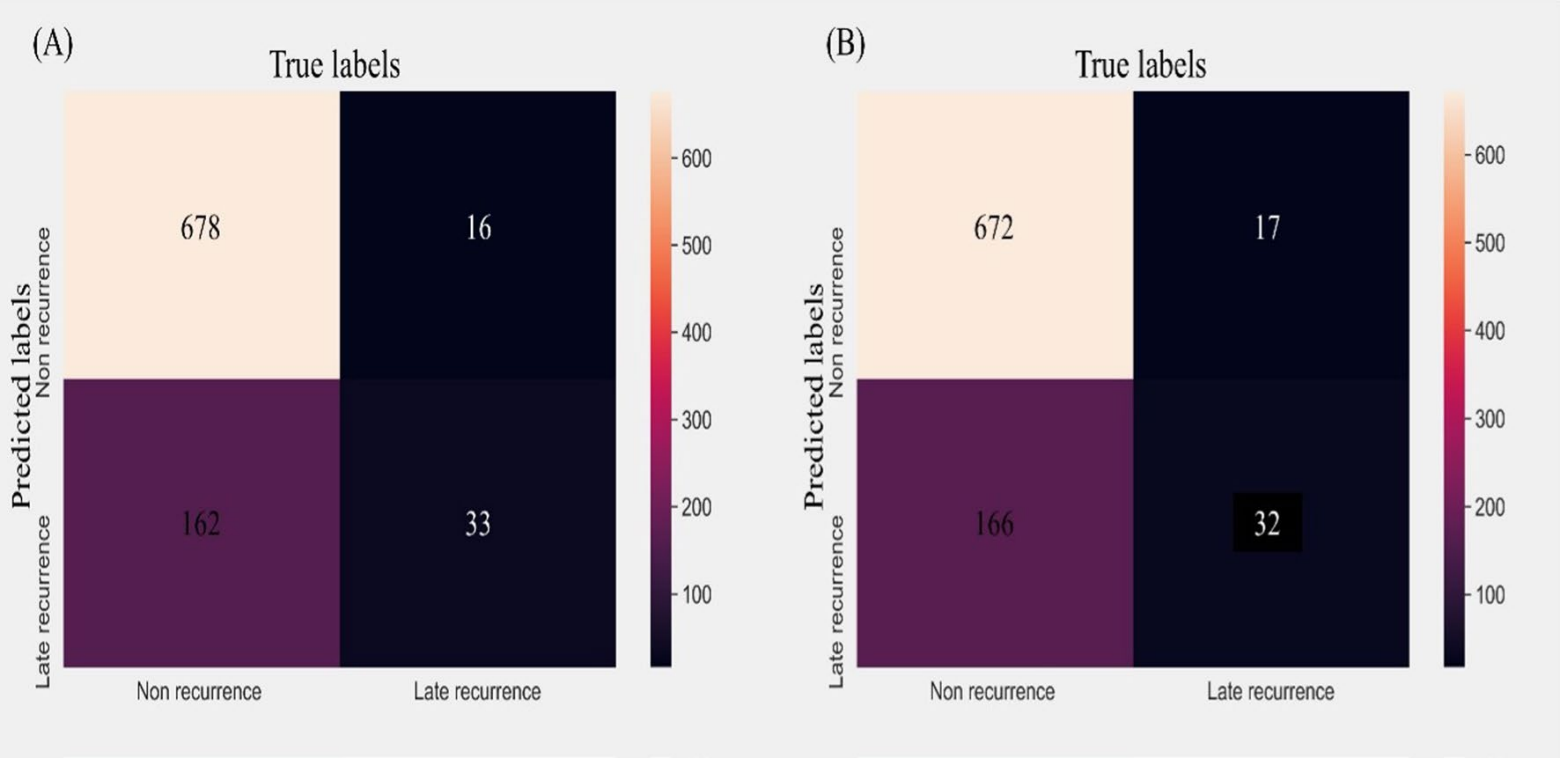
Confusion matrix of the top two performing models: **a** AdaBoost; **b** Logistic regression

The ROC curve for each model is shown in Fig. 2; AdaBoost has the highest AUROC (0.74). Next, performance was compared through the confusion matrix of AdaBoost, which had the highest performance, and logistic regression, which had the second-highest performance, as shown in Fig. 3. For FN, AdaBoost had 16, which was 1 less than for logistic regression (17), and for FP, AdaBoost had 162, which was 4 less than for logistic regression (166). In addition, when comparing the F1-score, which is the performance seen together with AUROC when data imbalance exists, the logistic regression model shows 0.599 performance, while Adaboost shows a higher 0.609 performance.

Thus, AdaBoost showed the highest performance in classification accuracy compared with the other models, and we finally developed an algorithm using AdaBoost. In order to use the Adaboost model developed by us, the user first inputs patient data (e.g., operation type, operative method, pathological tumor stage, pathological node stage, histologic type, lymphovascular invasion, tumor size). In the final selected hyperparameters (n_estimator = 500, learning_rate = 0.05, max_depth = 3), the Adaboost model returns a predicted value for the patient information input.

## Discussion

**Fig. 4:**
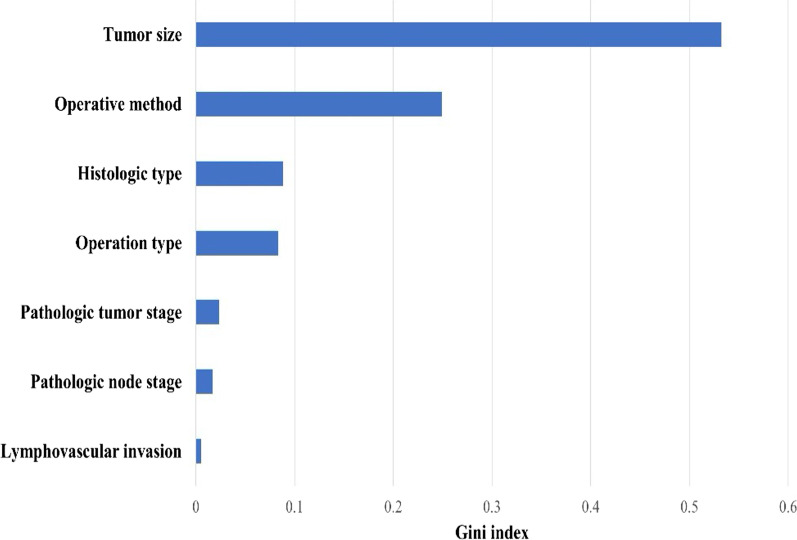
Importance of each variable in the AdaBoost model

In the current study, the data of 2956 patients with RCC collected from eight tertiary hospitals in Korea were analyzed. We successfully developed an algorithm to predict the likelihood of late recurrence in patients with RCC after surgery using seven clinicopathological factors. Of the eight machine learning models used, AdaBoost showed the best performance. Despite the powerful predictive ability of machine learning, the biggest drawback is that it is difficult for humans to interpret the final classification process through a complex structure [44]. However, tree-based models such as AdaBoost can measure feature importance using the Gini index [45] as it has the advantage of clarifying which variables have a high influence on prediction. The AdaBoost variable importance analysis showed that tumor size was the most important variable followed by surgery type, histologic type, operation type, pathological tumor stage, pathological node stage, and lymphovascular invasion, in that order (Fig. 4).

In patients with localized RCC, tumor size was shown to be significantly associated with survival and recurrence, with local recurrence-free survival significantly decreasing with each 1-cm increase in tumor size [46]. Our data showed that the average tumor size was significantly different between the late recurrence (68.2 mm) and non-recurrence (38.8 mm) groups. In the late recurrence group, the rates of radical nephrectomy and open surgery were 82% and 75.1%, respectively. However, in the non-recurrence group, similar rates were shown by radical nephrectomy, partial nephrectomy and by open and laparoscopic surgeries. In previous studies, patients with clear cell RCC had significantly poorer 5-year recurrence-free survival than patients with papillary RCC and chromophobe RCC (78% vs. 86% vs. 91%, *P* =  0.001) [47]. In our data, the clear cell ratio was 91.0% in the late recurrence group and 84.7% in the non-recurrence group, with a greater proportion in the late recurrence group. Pathologic tumor stage [12, 15, 18], pathologic node stage [17], and lymphovascular invasion [15], which had relatively low variable importance, have also been proven to be significant variables for late recurrence in various studies.

In previous studies, 10 variables (sex, age, body mass index, smoking status, pathological tumor stage, histologic type, necrosis, lymphovascular invasion, capsular invasion, and Fuhrman nuclear grade) were significant predictors of early recurrence [27]. However, seven variables (sex, age, body mass index, smoking, capsular invasion, Fuhrman nuclear grade, and necrosis) were non-significant (*P* > 0.05) in late recurrence prediction. Moreover, in our results, four variables (operation type, operative method, pathological node stage, and tumor size) were found to be significant variables for classifying both non-recurrence and late recurrence.

The 10-year recurrence prediction algorithm developed in the previous study included patients who recurred within 5 years [27]. In contrast, our study included only patients with RCC that recurred >5 years after surgery. Therefore it is difficult to directly compare the performance our new algorithms with those previously developed. Hence, a limitation of the study is that performance and time complex comparisons with related studies could not be carried out.

Although studies have been conducted to explore factors influencing late recurrence using statistical methods, our study is the first to develop a model that directly predicts late recurrence of RCC using machine learning. The reason for the scarcity of studies is that late recurrence occurs in only about 10% of cases, and after 5 years, necessitating a very long follow-up period; thus, it is difficult to collect sufficient data for machine learning analysis. In general, variable selection is applied after dataset splitting. However, in small datasets like ours, we found that significant variable selection varied depending on how the dataset was split. Considering this bias, we first selected variables using all data and then split the dataset. The KORCC group has continuously collected data from hospitals with the largest number of patients with RCC in Korea, and we were able to develop a predictive model using the collected data.

Since our data were collected from eight Korean hospitals, we present the results that reflect both internal and external verification. However, there is a limitation as we were unable to conduct additional external verification through the hospitals of other countries; our model was developed to suit the characteristics of patients with RCC in Korea. Therefore, performance cannot be guaranteed when applied to patients in other countries. Our model showed an AUROC of 0.74. An AUROC of 0.7 to 0.8 is indicative of an acceptable model, and if it exceeds 0.8, the model is considered excellent [48]. Machine learning shows better performance when provided with a large amount of data. In future studies, we aim to develop an excellent model with an AUROC ≥ 0.8 when more late recurrence patient data are available in the KORCC database.

## Conclusions

We successfully developed an algorithm to predict late recurrence using the AdaBoost model, a machine-learning technique. The developed predictive model calculates the risk of late recurrence for each patient based on the collected data. This algorithm should help clinicians select patients who need a long-term follow-up of ≥ 5 years after surgery and to design treatment plans accordingly. In the future, it is necessary to improve and stabilize the model performance through additional external validation studies using larger samples.

## Supplementary Information

**Additional file 1**. Results of the first variable selection process

## Data Availability

The datasets used and analyzed during the current study are available from the corresponding author on reasonable request.

## Abbreviations

AUROC: Area under the receiver operating characteristic curve
FN: The number of false non-recurrences
FP: The number of false-positive late recurrences
IRB: Institutional Review Board
KNN: K-nearest neighbor
KORCC: KOrean Renal Cell Carcinoma
NB: Naïve Bayes
RCC: Renal cell carcinoma
SMOTE: Synthetic minority oversampling technique
SVM: Support vector machine
TN: The number of true non-recurrences
TP: The number of true late recurrences
XGBoost: Extreme gradient boosting
