# Supplementary material for: Machine learning-based prediction model for late recurrence after surgery in patients with renal cell carcinoma
Source: BMC Med Inform Decis Mak. 2022 Sep 13;22:241. doi: 10.1186/s12911-022-01964-w (PMC9472380; doi:10.1186/s12911-022-01964-w)
Supplement: Supplementary file 1 — Additional file 1. Results of the first variable selection process [file 12911_2022_1964_MOESM1_ESM.docx]

**Additional File 1**

**Results of the first variable selection process (18 variables)**

| Variable | Late recurrence group  (189 Patients) | Non-recurrence group  (2,767 Patients) | *P*-value |
| --- | --- | --- | --- |
| Operation type |  |  | <0.001 |
| Radical nephrectomy | 155 (82.0%) | 1,336 (48.3%) |  |
| Partial nephrectomy | 34 (18.0%) | 1,431 (51.7%) |  |
| Operative method |  |  | <0.001 |
| Laparoscopic | 29 (15.3%) | 1,051 (38.0%) |  |
| HALS | 6 (3.2%) | 78 (2.8%) |  |
| Open | 142 (75.1%) | 1,221 (44.1%) |  |
| Robotic | 12 (6.3%) | 417 (15.1%) |  |
| Pathological tumor stage |  |  | <0.001 |
| 1a | 52 (27.5%) | 1,811 (65.4%) |  |
| 1b | 49 (25.9%) | 579 (20.9%) |  |
| 2a | 38 (20.1%) | 131 (4.7%) |  |
| 2b | 7 (3.7%) | 48 (1.7%) |  |
| 3a | 34 (18.0%) | 167 (6.0%) |  |
| 3b | 7 (3.7%) | 20 (0.7%) |  |
| 3c | 0 (0.0%) | 2 (0.1%) |  |
| 4 | 2 (1.1%) | 9 (0.3%) |  |
| Pathological node stage |  |  | 0.005 |
| Nx | 67 (35.4%) | 1,585 (57.3%) |  |
| N0 | 115 (60.8%) | 1,169 (42.2%) |  |
| N1 | 7 (3.7%) | 13 (0.5%) |  |
| Histologic type |  |  | 0.002 |
| Clear cell | 172 (91.0%) | 2,345 (84.7%) |  |
| Papillary | 5 (2.6%) | 41 (1.5%) |  |
| Chromophobe | 4 (2.1%) | 222 (8.0%) |  |
| Collecting duct | 0 (0.0%) | 3 (0.1%) |  |
| Etc. | 8 (4.2%) | 156 (5.6%) |  |
| Lymphovascular invasion |  |  | <0.001 |
| No | 172 (91.0%) | 2,701 (97.6%) |  |
| Yes | 17 (9.0%) | 66 (2.4%) |  |
| Tumor size (mm) | 68.2$\pm68.9$ | 38.8$\pm27$.6 | <0.001 |
| Creatinine | 1.0$\pm0.2$ | 1.2$\pm1$.3 | <0.001 |
| Albumin | 4.2$\pm0.4$ | 4.4$\pm0$.4 | <0.001 |
| Platelet | 248.8$\pm74.7$ | 233.4$\pm64$.4 | 0.014 |
| ESR | 20.6$\pm22.4$ | 14.9$\pm18$.0 | 0.018 |
| MDRD-GFR | 71.4$\pm16.3$ | 75.7$\pm26$.3 | 0.001 |
| CKD-EPI-GFR | 76.4$\pm16.9$ | 80.2$\pm20$.9 | 0.004 |
| Age | 55.8$\pm10.8$ | 54.1$\pm12$.3 | 0.0037 |
| Symptom: incidentaloma |  |  | <0.001 |
| No | 69 (36.5%) | 440 (15.9%) |  |
| Yes | 120 (63.5%) | 2,327(84.1%) |  |
| Symptom: hematuria |  |  | <0.001 |
| No | 155 (82.0%) | 2,584 (93.4%) |  |
| Yes | 34 (18.0%) | 183 (6.6%) |  |
| Symptom: flank pain |  |  | <0.001 |
| No | 159 (84.1%) | 2,588 (93.5%) |  |
| Yes | 30 (15.9%) | 179 (6.5%) |  |
| Necrosis |  |  | 0.042 |
| No | 165 (87.3%) | 2,512 (90.8%) |  |
| Microscopic | 14 (7.4%) | 190 (6.9%) |  |
| Macroscopic | 10 (5.3%) | 65 (2.3%) |  |

MDRD-GFR: modification of diet in renal disease glomerular filtration rate

CKD-EPI-GFR: chronic kidney disease epidemiology collaboration glomerular filtration rate

HALS: hand-assisted laparoscopic surgery
